# Key aspects of soft tissue management in fracture-related infection: recommendations from an international expert group

**DOI:** 10.1007/s00402-023-05073-9

**Published:** 2023-11-03

**Authors:** Leonard C. Marais, Sven Hungerer, Henrik Eckardt, Charalampos Zalavras, William T. Obremskey, Alex Ramsden, Martin A. McNally, Mario Morgenstern, Willem-Jan Metsemakers, the FRI Consensus Group, William T. Obremskey, Martin A. McNally, Bridget L. Atkins, Olivier Borens, Melissa Depypere, Kenneth A. Egol, Austin T. Fragomen, Jolien Onsea, Geertje A. M. Govaert, Stephen L. Kates, Richard Kuehl, Ian Mcfadyen, T. Fintan Moriarty, Michael Raschke, R. Geoff Richards, Carlos Sancineto, Eric Senneville, Andrej Trampuz, Michael H. J. Verhofstad, Werner Zimmerli

**Affiliations:** 1 Department of Orthopaedics, School of Clinical Medicine, University of KwaZulu-Natal, Durban, South Africa; 2 Department of Joint Surgery and Arthroplasty, Trauma Center Murnau, Murnau Germany and Paracelsus Medical University (PMU) Salzburg, Salzburg, Austria; 3 Department of Orthopaedic and Trauma Surgery, University Hospital Basel, Basel, Switzerland; 4 Department of Orthopaedic Surgery, Keck School of Medicine, University of Southern California, Los Angeles, USA; 5 Department of Orthopaedic Surgery and Rehabilitation, Vanderbilt University Medical Center, Nashville, TN USA; 6 The Bone Infection Unit, Nuffield Orthopaedic Centre, Oxford University Hospitals, Oxford, UK; 7 Department of Trauma Surgery, University Hospitals Leuven, Leuven, Belgium

**Keywords:** Fracture-related infection, Soft tissue closure, Soft tissue cover, Soft tissue defects, flap, Negative pressure wound therapy, Fasciocutaneous flap, Muscle flap, Fracture, Infection

## Abstract

A judicious, well-planned bone and soft tissue debridement remains one of the cornerstones of state-of-the-art treatment of fracture-related infection (FRI). Meticulous surgical excision of all non-viable tissue can, however, lead to the creation of large soft tissue defects. The management of these defects is complex and numerous factors need to be considered when selecting the most appropriate approach. This narrative review summarizes the current evidence with respect to soft tissue management in patients diagnosed with FRI. Specifically we discuss the optimal timing for tissue closure following debridement in cases of FRI, the need for negative microbiological culture results from the surgical site as a prerequisite for definitive wound closure, the optimal type of flap in case of large soft tissue defects caused by FRI and the role of negative pressure wound therapy (NPWT) in FRI. Finally, recommendations are made with regard to soft tissue management in FRI that should be useful for clinicians in daily clinical practice.

**Level of evidence** Level V.

## Introduction

Fracture-related infection (FRI) remains an important complication after musculoskeletal trauma, with an enormous impact on patients and healthcare systems [1, 2]. A meticulous well-planned and well-executed debridement remains one of the cornerstones of treatment, with the goal of removing all non-viable and/or infected tissue [3, 4]. All tissue that cannot contribute to wound and fracture healing, such as necrotic, ischaemic or scar tissue, needs to be excised and replaced with well-vascularized tissue [5]. The goal is to have only well perfused healthy tissue at the completion of debridement [6]. Up to 40% of FRI cases may require local, pedicled or free flaps to reconstruct the soft tissues [7]. Management of soft tissue defects that result from debridement is complex and numerous factors need to be considered when selecting the appropriate treatment strategy.

Ideal soft tissue cover can be defined as well vascularized, space filling, preferably sensate, epithelialized tissue that is soft, not fibrotic, robust and stable, covering bone and metal implants with optimal function and cosmetic appearance. The objective is to provide a robust barrier preventing further bacterial contamination of the fracture site and a biological environment conducive to fracture healing and eradication of infection whilst not getting in the way of a good functional outcome.

Numerous approaches are in use in the management of infection-related soft tissue defects. Traditionally, a staged approach was preferred with initial debridement followed by definitive soft tissue closure at a later stage [5]. More recently, definitive soft tissue closure has been performed at the same time as the debridement [8, 9]. The type of flap, be it muscle or fasciocutaneous, local or free, remains up for discussion. There has been a shift away from open bone grafting techniques in chronic osteomyelitis surgery, with an increased interest in single-stage reconstruction often including local antibiotic delivery [9–14]. Negative pressure wound therapy (NPWT) remains popular in the temporary management of soft tissue defects associated with open fractures as a bridge to final surgery. However, its role in established infection remains controversial.

This narrative review examines the current evidence with respect to soft tissue management in patients diagnosed with FRI. Specifically we discuss the optimal timing for tissue closure following debridement in cases of FRI, the need for negative microbiological culture results from the surgical site as a prerequisite for definitive wound closure, the optimal type of flap in case of large soft tissue defects caused by FRI and the role of negative pressure wound therapy (NPWT) in FRI. Furthermore, general recommendations are made regarding soft tissue management in FRI that should be useful for clinicians in daily clinical practice.

## Timing of soft tissue closure

There is a lack of evidence concerning the optimal timing of soft tissue closure in established FRI. There is, in fact, a shortage of data on the optimal approach to soft tissue management in FRI, in general. As a result, data from the open fracture literature related to the prevention of FRI will be presented, as it may offer some insights into the management of established FRIs. Of course, it must be recognized that the management of soft tissue defects in the setting of an established infection is somewhat different and often more challenging.

In open fractures, current clinical data suggest that definitive soft tissue closure should be achieved as soon as possible, optimally within one week, to decrease the risk of subsequent infection [15–25]. The move towards early soft tissue closure in open tibia fractures is epitomized in the “fix and flap” concept, where immediate soft tissue closure is provided [17, 26]. This approach, however, requires microsurgical teams being available on a permanent 24-h basis and may not be feasible in many centers. Furthermore, it is difficult to describe an exact time by which an open fracture needs to be covered with the currently available evidence [15]. The British Orthopaedic Association and British Association of Plastic, Reconstructive and Aesthetic Surgeons currently recommend that definitive soft tissue closure should be provided within 72 h of injury in monotrauma patients (BOAST 4 guidelines) [15]. Overall, guidelines appear to agree that closure should not be delayed beyond 1 week.

When considering that wounds associated with open fractures are always contaminated, some parallels may be drawn between soft tissue cover of bone following debridement in open fractures and FRIs. While the general approach may be somewhat similar, the nature of soft tissue defects in FRI is significantly different due to scar formation, chronic inflammation and previous surgical procedures. A delay in wound closure following debridement in FRI may result in prolonged exposure to pathogens from the hospital environment, increasing the risk for recurrence of infection or secondary reinfection [27]. In the past, a staged approach was preferred in FRI, with soft tissue closure not being performed at the same time as the initial debridement [28]. However, since the 1970s, treatment of infected non-unions and osteomyelitis with excision, external fixation and immediate flap reconstruction have shown positive outcomes [27, 29]. More recently, several reports have emerged with good results when soft tissue closure was performed as part of the index operation. McNally et al. reported 96% eradication of infection in 76 infected tibial non-union cases treated by Ilizarov treatment protocols and either primary skin closure or flap cover during the index surgical procedure [30]. Others have similarly reported success rates of over 90% in patients suffering from chronic osteomyelitis managed with debridement and primary soft tissue closure at the same time [31, 32].

Comparable success rates have, however, also been reported with the use of a short interval staged approach [33, 34]. For example, Patzakis et al. reported a 100% success in terms of infection control in 32 patients with a mean time of 4 days between the debridement and flap cover [33]. Nasser et al. recently compared the outcome of single and two-stage orthoplastic reconstruction in 96 patients with FRI and osteomyelitis. Patients managed with single stage reconstruction tended to have lower recurrence of infection, lower amputation rates and lower rates of fixation failure [35]. This study, as most studies on the topic, may be subject to potential selection bias. Complicated cases, with large soft tissue defects, non-healed fractures, difficult to treat infections or other risk factors for a complicated clinical course, may be more likely to be included in the staged procedure arm of studies.

Soft tissue closure at time of the index procedure has the benefits of a lower number of surgical procedures for the patient with an associated shorter hospital stay and a shortened exposure to possible contamination with hospital acquired bacteria. On the other hand, the staged approach may be needed for certain patient-related (e.g., sepsis) or logistical reasons.

In summary, the open fracture literature suggests that a delay in wound closure beyond one week increases the risk of infection. The few studies directly comparing a single versus staged approach to soft tissue reconstruction in FRI indicate that outcomes appear similar, therefore indirectly favouring single stage procedures as these result in a lower patient burden. Both are, however, viable options as long as early soft tissue closure is achieved, and the choice may be individualized based on the specific characteristics of each case and the logistics of the treating center. The evidence for this recommendation is weak and future appropriately powered studies are required for definitive guidance.

## The role of microbiological culture results as a prerequisite for wound soft tissue closure

The presence of bone infection prior to flap cover of lower extremity soft tissue defects is associated with an increased risk of adverse surgical outcomes when compared to cases without bone infection [36, 37]. Nevertheless, the importance of a negative culture prior to wound closure remains controversial. The differentiation between a contaminated and an infected wound is not obvious. There are only a few reports focusing on the role of negative or positive microbiology in wounds prior to soft tissue closure. In 1995, Breidenbach and Trager suggested that quantitative wound cultures may have value in predicting infection in complex extremity soft tissue defects closed by free flaps [38]. However, subsequent reports have failed to support this concept. For example, no correlation was found between the density of microorganisms in deep tissue and the eventual outcome of myocutaneous rotation flap surgery in pressure sores [39]. Similarly, Diefenbeck et al. found no correlation between bacterial load in tissue culture at time of final debridement and outcome in early post-operative infections [40]. In fact, there was a similar recurrence rate in patients with and without negative cultures at time of wound closure. Furthermore, in periprosthetic joint infections, debridement and implant retention (DAIR) and one-stage revision surgery can be performed with successful outcomes, suggesting the underlying infection can be eradicated, provided that a thorough debridement is performed [41–43]. Ultimately, there is currently no evidence indicating that negative cultures at the time of definitive wound closure in FRI improve long term outcome or decrease risk for persistent infection. In this context, the benefits of early wound closure, as described above, outweigh any possible risks. Delaying wound closure due to absence of negative cultures at time of closure can, therefore, not be recommended.

## Type of flap

**Fig. 1:**
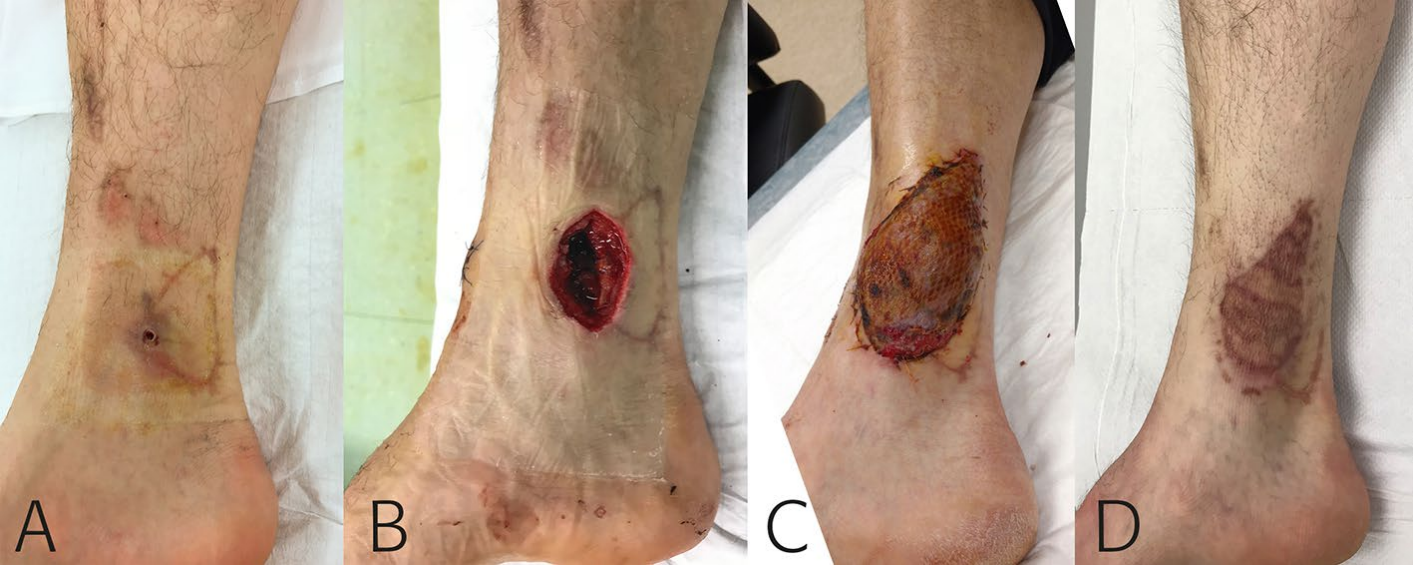
Fracture-related infection (FRI), caused by *Enterobacter cloacae,* in a 48 year-old male following intramedullary nailing of the tibia. **A** The patient presented with a draining fistula four months after the initial placement of the intramedullary nail. **B**, **C** After debridement a two-stage exchange of the nail was performed. The soft tissue defect was definitively closed with a free muscle (i.e. gracilis) flap. **D** One year postoperatively the flap had healed well and the patient remained infection free

**Fig. 2:**
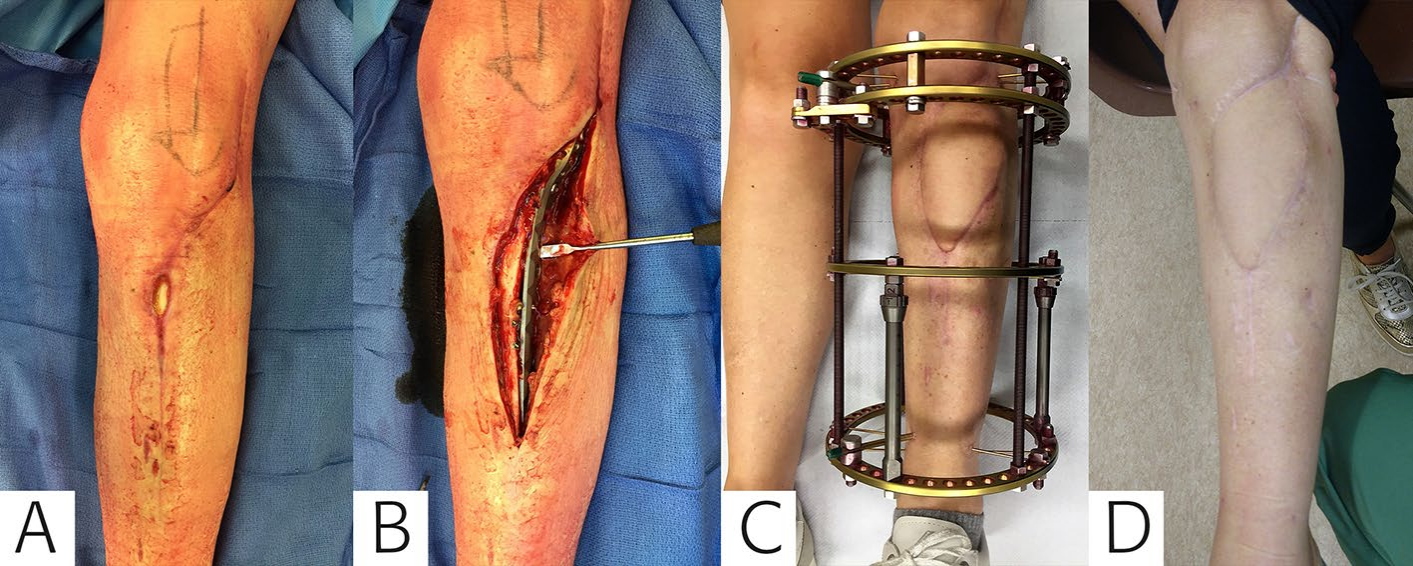
A polymicrobial fracture-related infection (FRI)*,* in a 45 year-old female following plate osteosynthesis of the tibia. **A** The patient presented with a draining fistula 2 weeks after the initial procedure. **B**, **C** After debridement and removal of all hardware (inadequate reduction and implant loosing) (first stage), a ring fixator was placed and the soft tissue defect was definitively closed with a transverse musculocutaneous gracilis (TMG) flap (second stage). **D** One year postoperatively the flap had healed well and the patient remained infection free

Numerous types of flaps may be used in cases of FRI when primary tension-free soft tissue closure is not possible (Figs. 1, 2). McNally et al. reported an improved success rate from 80.4 to 92.1% with a free tissue transfer in the tibia, as compared to tibias closed without a free flap (HR 0.38; 95% CI 0.14–1.0) [41]. Müller et al. found that free flaps were not associated with an increase of recurrent infection, non-union or flap failure when compared with other soft tissue reconstruction procedures [7].

The use of free fasciocutaneous and muscle flaps, as well as local rotational fasciocutaneous and muscle flaps has been described [45]. Muscle flaps have historically been reported to be superior to fasciocutaneous flaps in the management of open contaminated wounds. May et al. reported recurrence of infection in 4% of 96 patients with chronic traumatic bone wounds closed by free muscle tissue transfer [46]. Experimental studies have shown that muscle flaps improve blood flow to the bone [47, 48]. Furthermore, an animal study directly comparing muscle and fasciocutaneous flaps demonstrated superior bacterial suppression in the muscle group [48]. Another potential benefit of muscle flaps is their ability to reduce dead space resulting from the debridement [50, 51]. However, a multicenter outcome analysis comparing muscle and fasciocutaneous flaps found no difference in limb salvage rates in both acute and chronic trauma wounds [52]. Following a retrospective study of their own results in combination with a systematic review comparing muscle and fasciocutaneous flaps, Liu et al. concluded that muscle flaps and fasciocutaneous flaps had a comparable success rate in the reconstruction of chronic osteomyelitis defects in the lower limb [53]. Others have supported this view with similar findings [4, 54, 55]. Recently, Müller et al. found no difference in 58 FRI cases with respect to recurrence or persistence of infection, non-union or failure of soft tissue reconstruction in relation to the type of flap used [7]. Primary flap failure occurred in 12% of cases (7 of 58) and patients with a high Charlson Comorbidity Index where at increased risk. Interestingly, the authors also showed that in case of primary flap failure within the first three weeks, salvage with secondary soft tissue reconstruction was feasible in all cases and failure was not associated with an increased risk for the development of non-union or recurrent infection [4].

Fallico et al. performed a review of clinical data on the outcome and complication rates with free and pedicled flaps used in cases with exposed hardware [55]. Overall, no significant difference was found in terms of flap survival. There was, however, an increased rate of successful implant retention (78 vs 53%) with pedicled flaps. This was attributed to selection bias, namely the fact that pedicled flaps may have been reserved for less complicated cases with less extensive soft tissue deficits or certain anatomic locations. Furthermore, the complication rate was 47% with pedicled flaps in comparison with only 10% with free flaps. The LEAP study group also found that in severe open tibia fractures, wound complications requiring operative intervention were much more (4.3 times) likely in patients treated with pedicled flaps compared to free flaps [56]. However, Hoyt et al. found that an increase in the annual volume of pedicled flaps for limb salvage was associated with a decrease in flap failure and reoperation rate [57].

Cho et al. compared muscle and fasciocutaneous free flaps in 518 post-traumatic lower extremity reconstructions (238 acute trauma cases and 280 chronic post-traumatic complication cases which included infections) [52]. The two types of flaps were found to be comparable in terms of limb salvage rate and functional recovery. The recurrence rate of infection was, however, not specified. Notably, in patients with Gustilo-Anderson (GA) type IIIB injuries, fasciocutaneous flaps were more commonly re-elevated for subsequent orthopaedic procedures like bone grafting or revision of hardware. Contrary to this, Hoyt et al. found an increased risk of flap failure with the use of muscle flaps in 330 limb salvage procedures [57]. Another consideration is bony healing and recently a study by Mehta et al. revealed that patients with acute GA type IIIB tibia fractures who received muscle flaps had significantly faster radiographic progression of bone healing in the first 6 months compared to patients who received fasciocutaneous flaps [58]. This is supported by the finding of Cho et al. that in a subgroup of patients with GA type III injuries fractures covered with fasciocutaneous flaps required more orthopedic interventions [52]. Although it should be noted that GA type IIIB injuries are not entirely comparable to FRI, the high contamination grade of these severe open fractures still has similarities to infected cases which are the focus of this review.

**Fig. 3:**
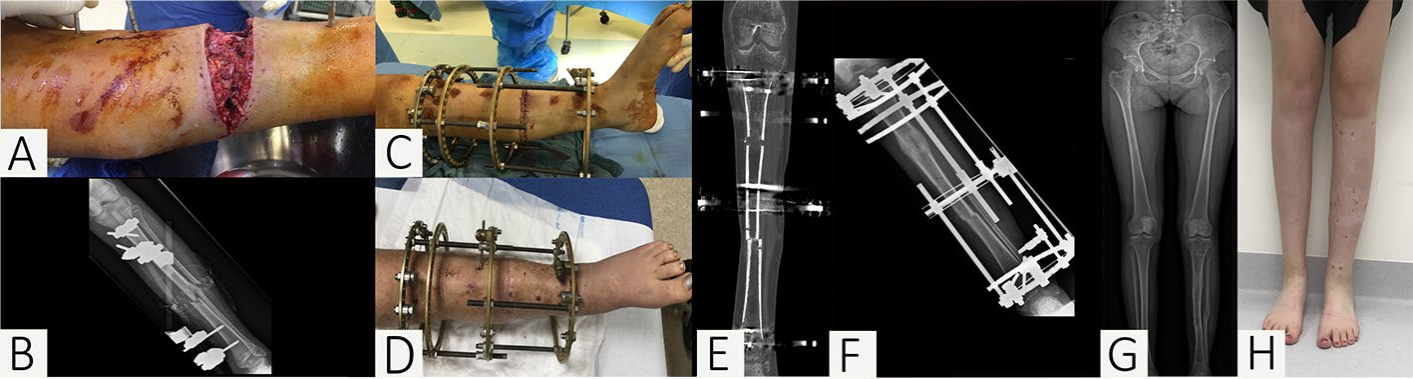
A culture-negative fracture-related infection (FRI) of the tibia in a 15-year-old polytrauma patient. Among multiple injuries, she sustained a Gustilo-Anderson type IIIB open tibia and fibula fracture of the left lower limb. She was admitted to a tertiary referral hospital 3 weeks after temporary fixation of the fractures with an external fixator. She was taken to the operating room due to an increase in serum inflammatory markers combined with purulent drainage from the wound under broad-spectrum antibiotic therapy. **A**, **B** A clinical image of wound taken during surgical debridement and an anteroposterior radiograph of the left lower limb showing the primary external fixation. **C**, **D** The wound was debrided, the tibia acutely shortened with the placement of a stable circular tensioned wire external ring fixator and the wound closed. A proximal corticotomy was performed to lengthen the tibia. Follow-up was uneventful, and local and systemic signs of infection disappeared within 2 weeks after the final surgery. The culture results remained negative. **E**, **F** A coronal CT-scan and an anteroposterior standard radiograph show the lengthening process of the proximal tibia. **G**, **H** An anteroposterior radiograph and a clinical image of the lower limbs 2 years after the injury, illustrating satisfactory wound healing with no clinical signs of infection. Moreover, the standard radiographs illustrate consolidation of the fracture and the proximal distraction site (restoring length and axis)

Aside from flap cover, there are other soft tissue reconstruction techniques which may also be considered. McNally et al., for example, described the use of acute compression (shortening) in infected tibial nonunions with defects of up to 5 cm (Fig. 3) [30]. There has recently also been interest in the use of intentional deformation to aid closure of complex tissue defects in open tibial fractures [59, 60].

Overall, it appears that muscle and fasciocutaneous free flaps have comparable results in the reconstruction of soft tissue defects and that the most important factor is prompt and robust wound closure. One might hypothesize that perfusion of the flap—as a proxy for its ability to deliver any factor that helps to eradicate the infection—is more important than its anatomical origin. Pedicled flaps may be an option in selected cases, although an increased complication rate needs to be considered. In case of early infections, there may be a benefit in the use of muscle flaps in the lower leg due to the potential for faster progression of fracture union.

## Role of negative pressure wound therapy (NPWT)

Negative pressure wound therapy (NPWT) has increasingly been used over the past decades in acute and chronic wound care. It was initially proposed as a definitive treatment for superficial skin ulcers and wounds. Over time, more and more reports indicate NPWT serves merely as a dressing during the period of open wound management, prior to definitive closure in open fractures, and not as a ‘therapy’ itself. Currently, most of the available scientific evidence focuses on its use in open fractures. Limited data is available with respect to the safety and efficacy of NPWT in the treatment of FRI.

Earlier studies suggested promising results with this type of ‘temporary’ wound dressing in the prevention of infection in open fractures [61]. In 2009, Stannard et al. showed that the use of NPWT was superior to conventional gauze dressings in terms of prevention of infection in patients with high-energy open fractures [62]. A systematic review, published in 2015 by Schlatterer et al. confirmed this finding. [63]. Later, however, in 2018, the WOLLF multicenter randomized trial, showed no reduction of the infection rate in open tibial injuries treated with NPWT compared to conventional sealed dressings [64]. In contrast, data has emerged suggesting that NPWT may be associated with an increased risk of infection. In response to concerns raised about NPWT facilitating superinfection, Bhandari and co-workers performed a post-hoc analysis of 2551 open fractures of which 331 underwent NPWT during the FLOW trial [23, 24]. They found that NPWT was associated with an increase in infection rate by a factor of 1.5–5.3 (depending on the GA type). A recent meta-analysis in open fractures showed no significant reduction in FRIs with the use of NPWT when compared to conventional management [65]. Ultimately, caution is needed when interpreting these findings as there is considerable lack of conformity in terms of treatment protocols and there are numerous confounding variables that are frequently not reported. It should be emphasized that the time to definitive closure is likely to impact the results and this is often not uniformly reported. The increased rates of infection following NPWT may be associated with its use as a substitute for early soft tissue cover, rather than with the procedure itself. To illustrate this presumption, Bhattacharryya et al. showed that the routine use of NPWT as a dressing after initial wound debridement does not lessen the need for prompt close open tibia fractures [22]. In cases with a delay in soft tissue closure of more than 7 days, the infection rate was 57% as compared to 12.5% in the early closure group. Another meta-analysis looking at GA type IIIB/C fractures noted a tendency of increased infectious risk with extended NPWT if it was associated with a delay of definitive soft tissue closure of more than 7 days [66].

As previously stated, less is known about the role of NPWT with respect to the management of soft tissue defects in established FRI. A few studies, with small case numbers, have mentioned the use of NPWT as a bridge to definitive microsurgical reconstruction of the soft tissue envelope [67]. A recent systematic review by Haidari et al., including eight studies (six cohort studies and two case–control studies) found no clear scientific evidence to support the use of NPWT as definitive soft tissue treatment in FRI [68]. The average reported duration of NPWT was 1.5 to 7 weeks. The highest rated paper in terms of the quality of the evidence in this review, published a rather high recurrence rate of 35% [67]. Haidari et al. concluded that NPWT may be used for a few days until definitive flap cover can be performed, and recommended that comparative studies should be performed to determine the role and safety of NPWT in the treatment of FRI. In a second systematic review on this topic by Jensen et al., seven of the ten included studies were case reports with less than ten patients [69]. The authors pointed out that the quality of the existing literature was low. Thus, no recommendation on the use of NWPT could be made.

More recently, three studies have specifically addressed this topic of NPWT in FRI. Hellebrekers et al. found that the use of NPWT following debridement was associated with lower success rates [70]. Treatment failure, which was defined as absence of fracture union and/or the presence of signs of infection, occurred in 56% of cases managed with NPWTs compared to 23% without the use of NPWT (*p* = 0.03). A cohort study, by Sweere et al. showed that the risk of recurrence more than doubles with the use of NPWT in FRI [71]. When comparing 99 patients who received NPWT for a median 18 days with 164 patients who were managed without, the authors noted recurrence of infection in 28% of the NPWT group in comparison with 12% in the control group (*p* = 0.001, 95% CI 0.174–0.635). In their multivariable model, the duration of NPWT emerged as an independent risk factor for the recurrence of infection. Notably, in this study all patients who underwent NWPT prior to the diagnosis of FRI (for example, to cover the wound of an initially open fracture) were excluded. Similarly, McNally et al. showed that recurrence of infection or unplanned treatment for possible infection occurred in 32% of cases where NPWT was used before skin closure, in comparison with 12% where it was not (HR 3.5; 95% CI 1.852–6.512) [44].

We should, therefore, be cautious in using NPWT in treatment pathways for FRI. Furthermore, the term NPWT is misleading as it should not be seen as a definitive type of wound treatment or “therapy”, but rather as a temporary type of ‘wound dressing’. Generally, wound healing by secondary intention is discouraged in FRI as it takes longer than primary wound healing (with subsequent risk of re-contaminating the bone) and because of the fact that the scar tissue that forms is poorly vascularized and of poor quality, which may result in recurrence of infection. In other words, the dense scar tissue that results from NPWT does not fully satisfy the definition of the optimal soft tissue closure and may be inadequate as an effective barrier against bacteria. In addition, if secondary procedures are required to secure fracture union, correct deformity or leg length, this poor tissue may prevent safe surgical approaches. The evidence from the use of NPWT in open fractures suggests that there is no strong justification for the extra cost, compared to standard dressings. Another concern relates to the high recurrence of infection and the potential for increased bacterial colonization [71, 72]. Mouës et al. showed that NPWT does not necessarily cause a reduction in bacterial load, but may shift the bacterial pathogen profile from Gram-negative rods towards *Staphylococcus aureus* [73]. Yusuf et al. showed in a study of 68 NPWT foams that bacteria persisted despite regular changes and new bacterial species appeared in over one quarter of cases [74].

In specific scenarios where multiple procedures are planned or where logistic issues prohibit definitive closure of the wound at the initial setting, NPWT may be considered a bridge to definite wound closure, without inducing a delay. This would be in line with the recommendations made by the International Expert Panel on NPWT who recommended that in open fractures NPWT should be stopped as soon as surgical closure is possible [75]. These periods should always be kept as short as possible, with maximum duration of about seven days [76]. The use of NPWT as a form of definitive soft tissue closure technique in FRI cases where implants are retained should be discouraged. It should be kept in mind that it is generally recommended to perform changes of the NPWT dressings every 3 to 5 days. Finally, in cases of FRI, NPWT dressing changes on the ward or as an outpatient procedure should be avoided and must be performed under aseptic conditions in an operating room and proper analgesia.

## Conclusion and recommendations

In summary, scientific evidence in the field of soft tissue reconstruction in association with FRI is still scarce. A multidisciplinary team approach, with a focus on individualized patient care, is crucial. A treatment pathway should be developed that includes early definitive soft tissue closure following extensive debridement with robust histopathological and microbiological sampling, and the establishment of bone stability. Key recommendations to such an approach are presented in Table 1. There appears to be no clear advantage in using a short interval staged approach over soft tissue closure at the time of the index procedure, but both approaches are valid as long as early definitive soft tissue reconstruction is achieved. Negative tissue cultures are not essential prior to wound closure. Furthermore, broad spectrum empiric antibiotic therapy should be instituted immediately following the debridement to prevent new biofilm formation [77]. In terms of flap choice, it appears that the decision should be individualized based on size and site of the defect, the availability of recipient vessels, the general condition of the patient and availability of a microvascular team. If total or partial flap failure occurs, salvage with secondary soft tissue reconstruction is feasible. The long-term use of NPWT in FRI should be strongly discouraged. While it may have a role as a *short-term* (less than 1 week) wound dressing method following debridement in FRI cases where immediate soft tissue reconstruction is not feasible, prompt definitive wound closure with well vascularized soft tissue is still the method of choice.

**Table 1:** Key recommendations

- The management of soft tissue defects that result from debridement is complex and numerous factors need to be considered when selecting the appropriate treatment strategy
- A multidisciplinary team approach is recommended to ensure rapid restoration of an effective local antibacterial barrier
- A delay of more than one week in soft tissue coverage following debridement may increase the risk for recurrent infection
- There appears to be no clear advantage in using a short interval staged approach over soft tissue closure at the time of the index procedure, but both approaches are valid as long as early definitive soft tissue reconstruction is achieved
- Negative tissue cultures from the debrided wound is not a prerequisite for definitive soft-tissue closure
- Muscle and fasciocutaneous (free and pedicled) flaps seem to have comparable results for reconstruction of soft tissue defects in FRI
- There may be a benefit to using muscle flaps in early infections in terms of fracture union
- The term NPWT is misleading and it should not be seen as a definitive type of wound treatment or “therapy” but rather as a temporary type of ‘wound dressing’
- NPWT should be seen as a bridge to definite soft tissue coverage and periods of NPWT use should always be kept to a minimum
FRI, fracture-related infection; NPWT, negative-pressure wound therapy
